# Supplementary material for: Interdomain-linkers control conformational transitions in the SLC23 elevator transporter UraA
Source: Nat Commun. 2024 Aug 30;15:7518. doi: 10.1038/s41467-024-51814-8 (PMC11362169; doi:10.1038/s41467-024-51814-8)
Supplement: Supplementary file 1 — Supplementary Information [file 41467_2024_51814_MOESM1_ESM.pdf]

Interdomain-linkers control conformational transitions in the SLC23 elevator transporter UraA – Supplementary Information

Supplementary Table 1: Monomer-dimer ratio of decylmaltoside-solubilized UraA variants as determined by size exclusion chromatography. Shown are average values and corresponding standard deviations of technical replicates (n=3 for all samples). The fraction of protomers migrating as monomers and dimers was calculated following peak deconvolution using Origin.

UraA variant	Monomer (%)	Dimer (%)
WT	79.2 ± 0.01	20.8 ± 0.01
G112P	80.2 ± 0.02	19.8 ± 0.02
G320P	55.1 ± 0.02	44.9 ± 0.02
P330G	17.6 ± 0.02	82.4 ± 0.02

Supplementary Table 2: Data collection and refinement statistics

	UraA(G320P)-Sy45	UraA(G320P)-Sy45 Uracil
**Data collection**		
Space group	P 1 21 1	P 1 21 1
Cell dimensions		
a, *b*, *c* (Å)	87.14 118.11 94.93	86.25 116.82 94.3
α, β, γ (°)	90 100.424 90	90 100.485 90
Resolution (Å)	29.53 - 3.50 (3.63 - 3.50)	48.10 - 3.70 (3.83 - 3.70)
R_{merge}	0.06798 (0.5788)	0.1407 (0.5994)
$I / \sigma I$	21.84 (5.72)	11.39 (3.86)
Completeness (%)	98.11 (86.32)	97.89 (81.54)
Redundancy	13.6 (13.7)	6.8 (6.6)
**Refinement**		
Resolution (Å)	3.5	3.7
No. reflections	23545 (2045)	19370 (1603)
R_{work} / R_{free}	0.2408/0.2599	0.2711/0.2944
No. atoms		
Protein	7969	7935
Ligand/ion	378	219
B-factors		
Protein	67.33	52.19
Ligand/ion	69.98	47.94
R.m.s. deviations		
Bond lengths (Å)	0.001	0.002
Bond angles (°)	0.39	0.45

*Values in parentheses are for highest-resolution shell.

Supplementary Table 3: Filter setting in DynamX

Parameter	Value
Minimum intensity	0
Minimum sequence length	3
Maximum sequence length	20
Minimum products	2
Minimum products per amino acid	0.1
Minimum consecutive products	0
Minimum sum intensity for products	0
Minimum score	6
Maximum MH+ Error (ppm)	0
File threshold	6
Retention time RSD	0
Intensity RSD	0

Supplementary Table 4: HDX summary table reporting critical HDX-MS information for the analysis of wild type UraA in the presence and absence of uracil.

Data Set	Control (WT)	Binding (WT+100 μM Uracil)
HDX reaction details	20 mM Hepes, 150 mM NaCl, 0.2% DM at pH 7.5	20 mM Hepes, 150 mM NaCl, 100 μM uracil, 0.2% DM at pH 7.5
HDX time course [seconds]	0, 30, 360, 900, 2700	0, 30, 360, 900, 2700
HDX control samples	-	-
Back-exchange (mean / IQR)	-	-
# of Peptides	153	
Sequence coverage	81.5%	
Average peptide length / Redundancy	10.40 / 4.33	
Replicates (biological or technical)	4 (technical)	4 (technical)
Repeatability	1.037 (Average RSD, exact values in Supplementary Data 2)	0.803 (Average RSD, exact values in Supplementary Data 2)
Significant differences in HDX (delta HDX > X D)	Peptide specific Two-staged T-test; first stage p≤0.05, second stage p≤0.01	

Supplementary Table 5: HDX summary table reporting critical HDX-MS information for the analysis of UraA(G320P) in the presence and absence of uracil.

Data Set	Control (UraA-G320P mutant)	Binding (UraA-G320P mutant + 100 µM Uracil)
HDX reaction details	20 mM Hepes, 150 mM NaCl, 0.2% DM at pH 7.5	20 mM Hepes, 150 mM NaCl, 100 µM uracil, 0.2% DM at pH 7.5
HDX time course [seconds]	0, 30, 360, 900, 2700	0, 30, 360, 900, 2700
HDX control samples	-	-
Back-exchange (mean / IQR)	-	-
# of Peptides	227	
Sequence coverage	90.2%	
Average peptide length / Redundancy	9.42 / 5.28	
Replicates (biological or technical)	4 (technical)	4 (technical)
Repeatability	0.797 (Average RSD, exact values in Supplementary Data 3)	0.548 (Average RSD, exact values in Supplementary Data 3)
Significant differences in HDX (delta HDX > X D)	Peptide specific Two-staged T-test; first stage p≤0.05, second stage p≤0.01	

Supplementary Table 6: HDX summary table reporting critical HDX-MS information for the analysis of UraA(P330G) in the presence and absence of uracil.

Data Set	Control (UraA-P330G mutant)	Binding (UraA-P330G + 100 µM Uracil)
HDX reaction details	20 mM Hepes, 150 mM NaCl, 0.2% DM at pH 7.5	20 mM Hepes, 150 mM NaCl, 100 µM uracil, 0.2% DM at pH 7.5
HDX time course [seconds]	0, 30, 360, 900, 2700	0, 30, 360, 900, 2700
HDX control samples	-	-
Back-exchange (mean / IQR)	-	-
# of Peptides	199	
Sequence coverage	89.2%	
Average peptide length / Redundancy	10.79 / 5.13	
Replicates (biological or technical)	4 (technical)	4 (technical)
Repeatability	0.637 (Average RSD, exact values in Supplementary Data 4)	0.529 (Average RSD, exact values in Supplementary Data 4)
Significant differences in HDX (delta HDX > X D)	Peptide specific Two-staged T-test; first stage p≤0.05, second stage p≤0.01	

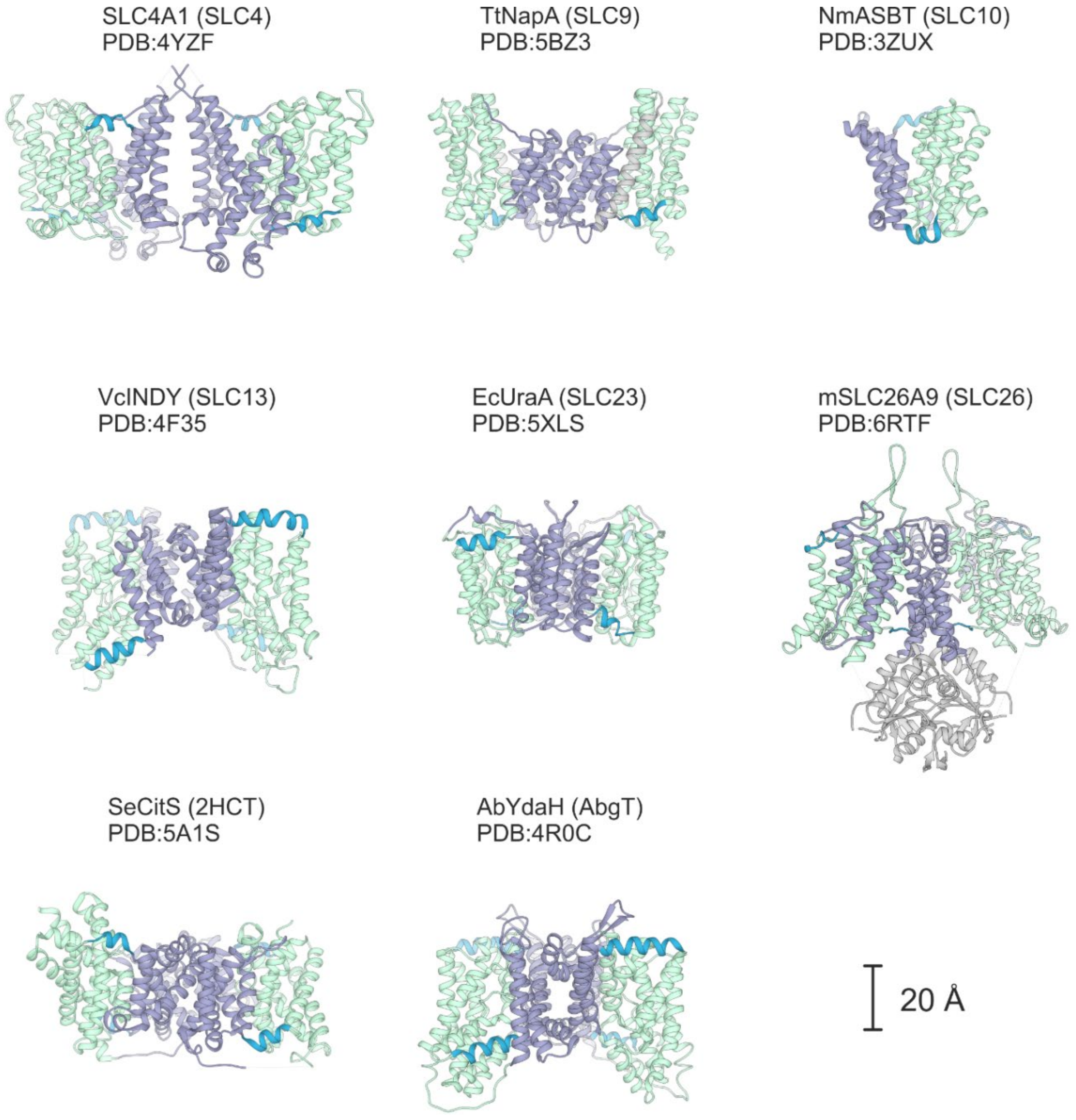

Supplementary Fig. 1: Elevator transporter families with spacer helix in the interdomain-linker. Side view on elevator transporter structures of the SLC4, 9, 10, 13, 23, 26, 2HCT and AbgT family. Scaffold and transport domains are shown in purple and green color, respectively and additional transmembrane segments or soluble domains in grey. Spacer helices are highlighted in blue.

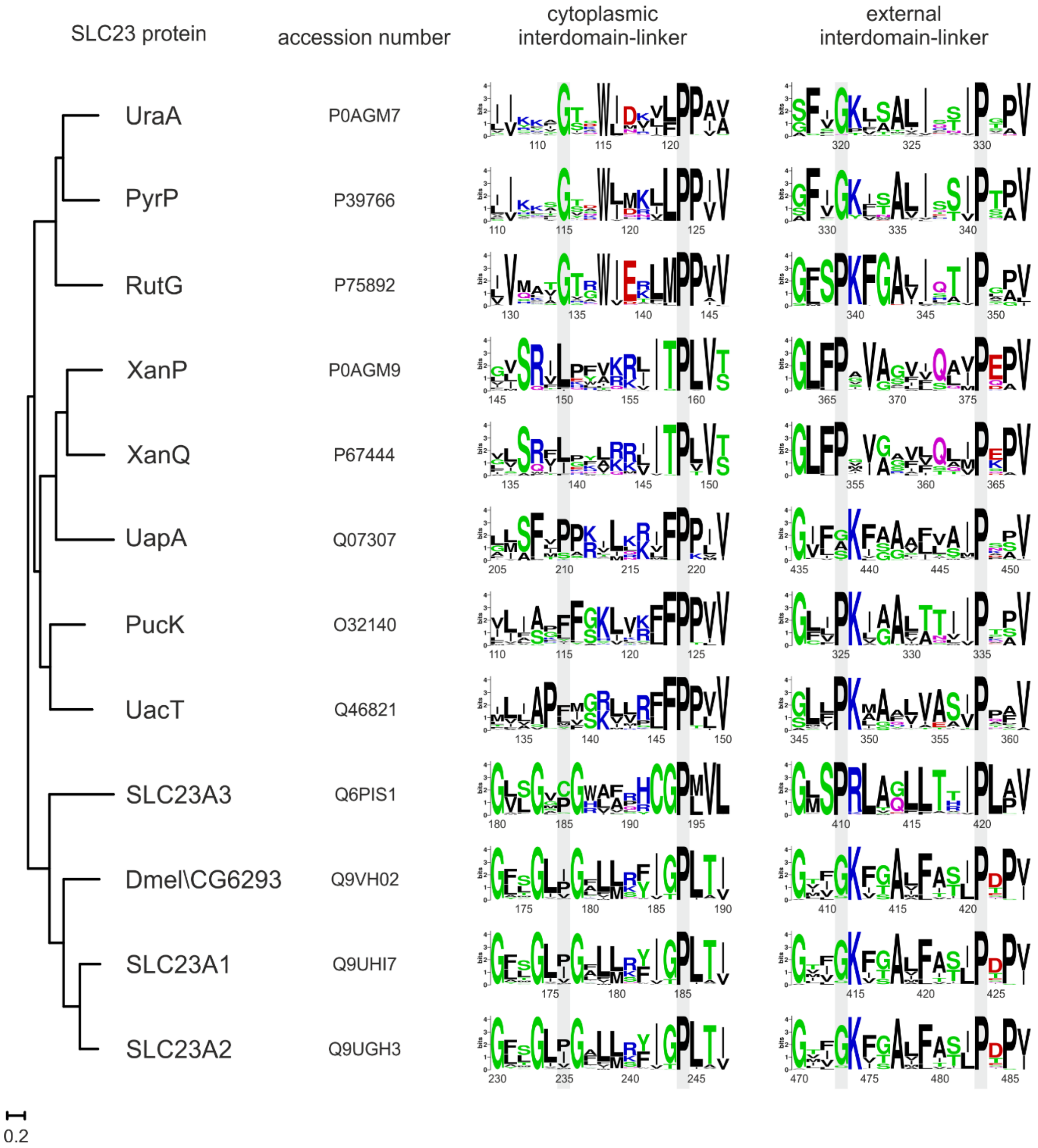

Supplementary Fig. 2: Phylogenetic analysis of inter-domain linkers in SLC23 family. Phylogenetic tree of SLC23 family members with uniprot accession numbers indicated (left panel). Alignment of cytoplasmic or external interdomain-linker sequences, respectively, from different SLC23 subfamilies (right panel). Subfamily members were identified by BLAST search with minimum sequence identity of 40% and minimum sequence coverage of 50% with respect to the query sequence.

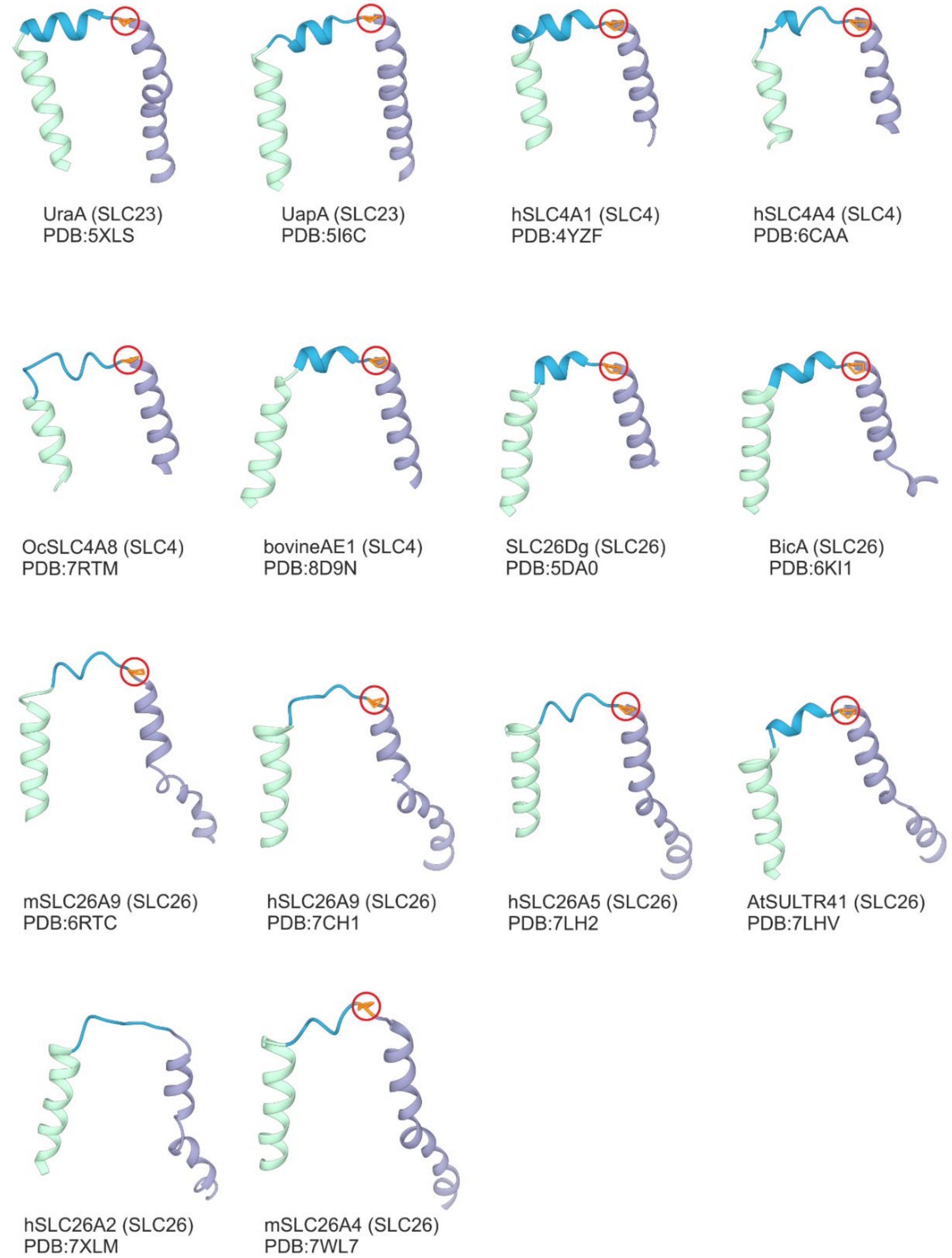

Supplementary Fig. 3: Extracellular interdomain-linker in 7-transmembrane segment inverted repeat (7TMIR) fold proteins of the SLC4, SLC23 and SLC26 family.
The extracellular interdomain-linker (blue) connects TM11 (green) of the transport domain and TM12 (purple) of the scaffold domain. The conserved proline residue at the scaffold domain facing side of the interdomain-linker and its similar side chain orientation is presented in stick representation (orange) and highlighted by a red circle.

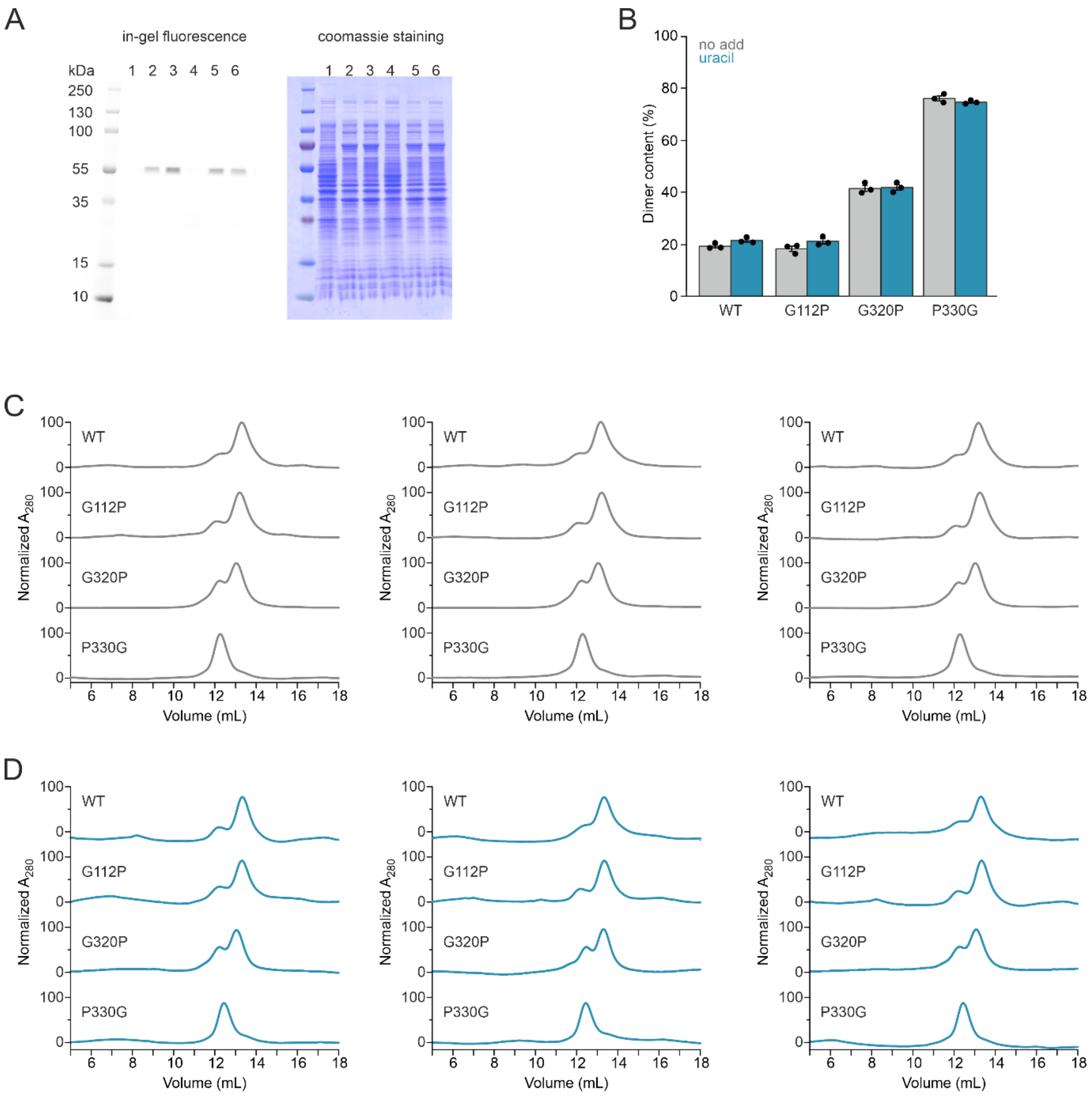

Supplementary Fig. 4: (A) In-gel fluorescence (left panel) and coomassie staining (right panel) of the same SDS-PAGE gel loaded with the samples 1: control (UraA(WT) uninduced); 2: UraA(WT); 3: UraA(G112P); 4: UraA(P121G); 5: UraA(G320P) and 6: UraA(P330G) expressed from the pBXC3GH plasmid as C-terminal GFP fusion protein. **(B)** Dimer content of decylmaltoside-solubilized UraA variants in absence and presence of 1 mM uracil based on triplicate size-exclusion analysis as shown in panel C and D. The dimer content is defined as the ratio of the area under the dimer peak and the total area under the curve derived from Gaussian peak deconvolution. **(C)** Size-exclusion chromatograms of decylmaltoside-solubilized UraA variants in absence of uracil. **(D)** Size-exclusion chromatograms of decylmaltoside-solubilized UraA variants in presence of 1 mM uracil.

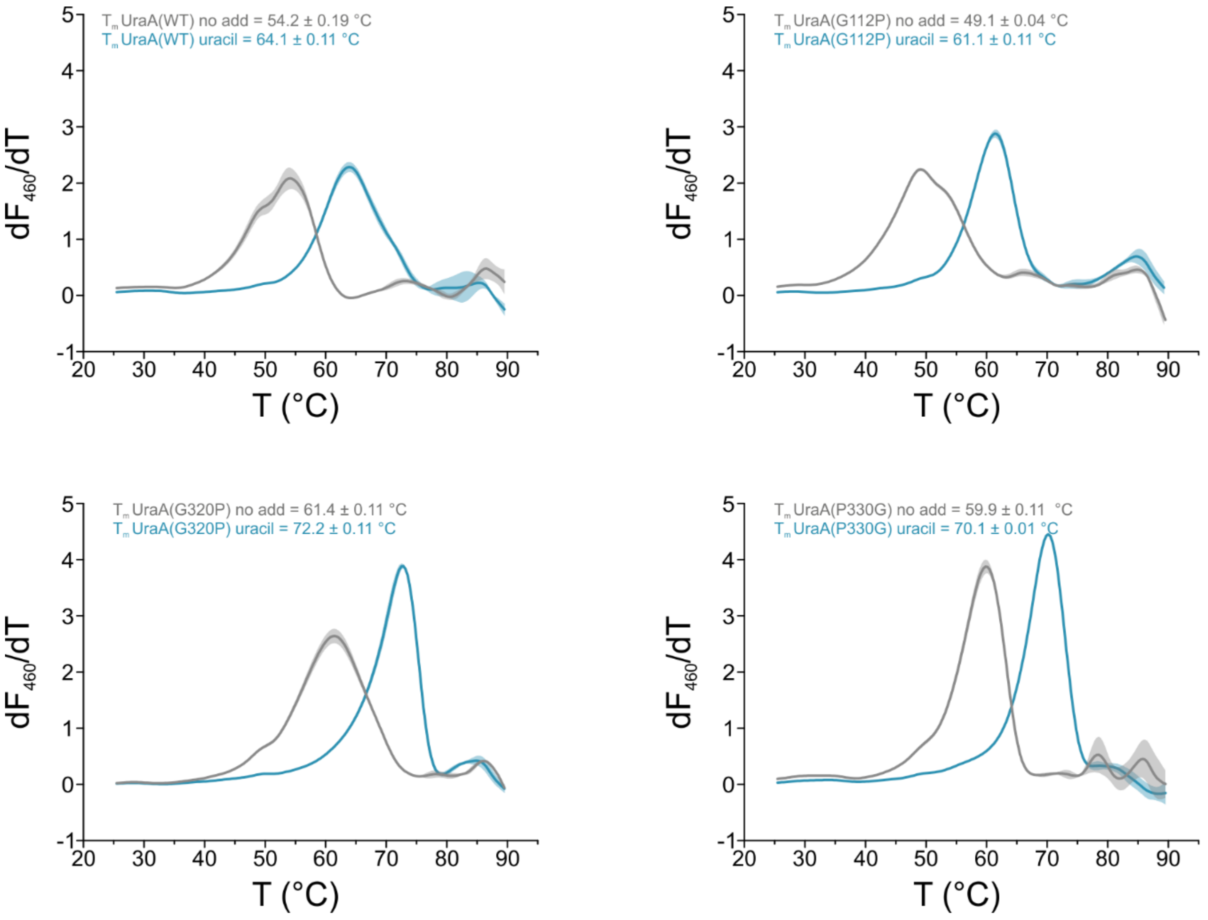

Supplementary Fig. 5: Differential scanning fluorimetry analysis of UraA(WT) and variant G112P, G320P and P330G as indicated in presence (blue) or absence (grey) of uracil. Shown are the mean of three technical replicates and the standard deviation.

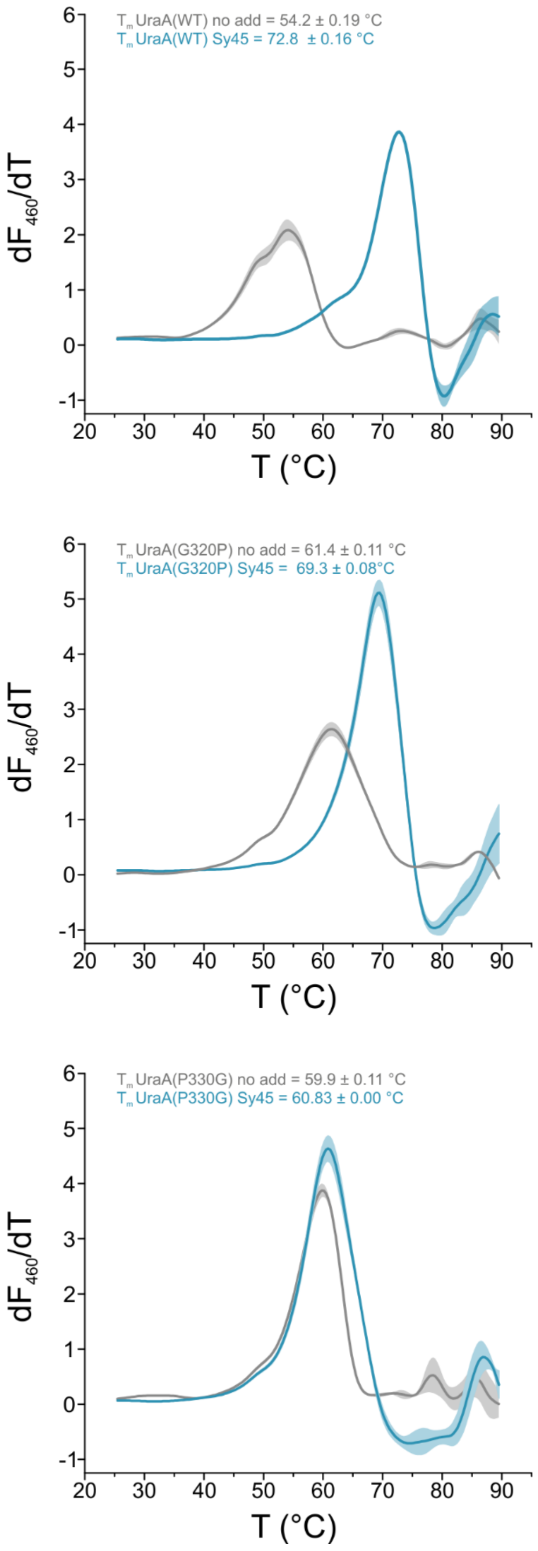

Supplementary Fig. 6: Differential scanning fluorimetry analysis of UraA(WT), UraA(G320P) and UraA(P330G) as indicated in presence (blue) or absence (grey) of the conformational probe Sy45. Shown are the mean of technical replicates (n=3 for no add samples; n=4 for Sy45 samples) and the standard deviation.

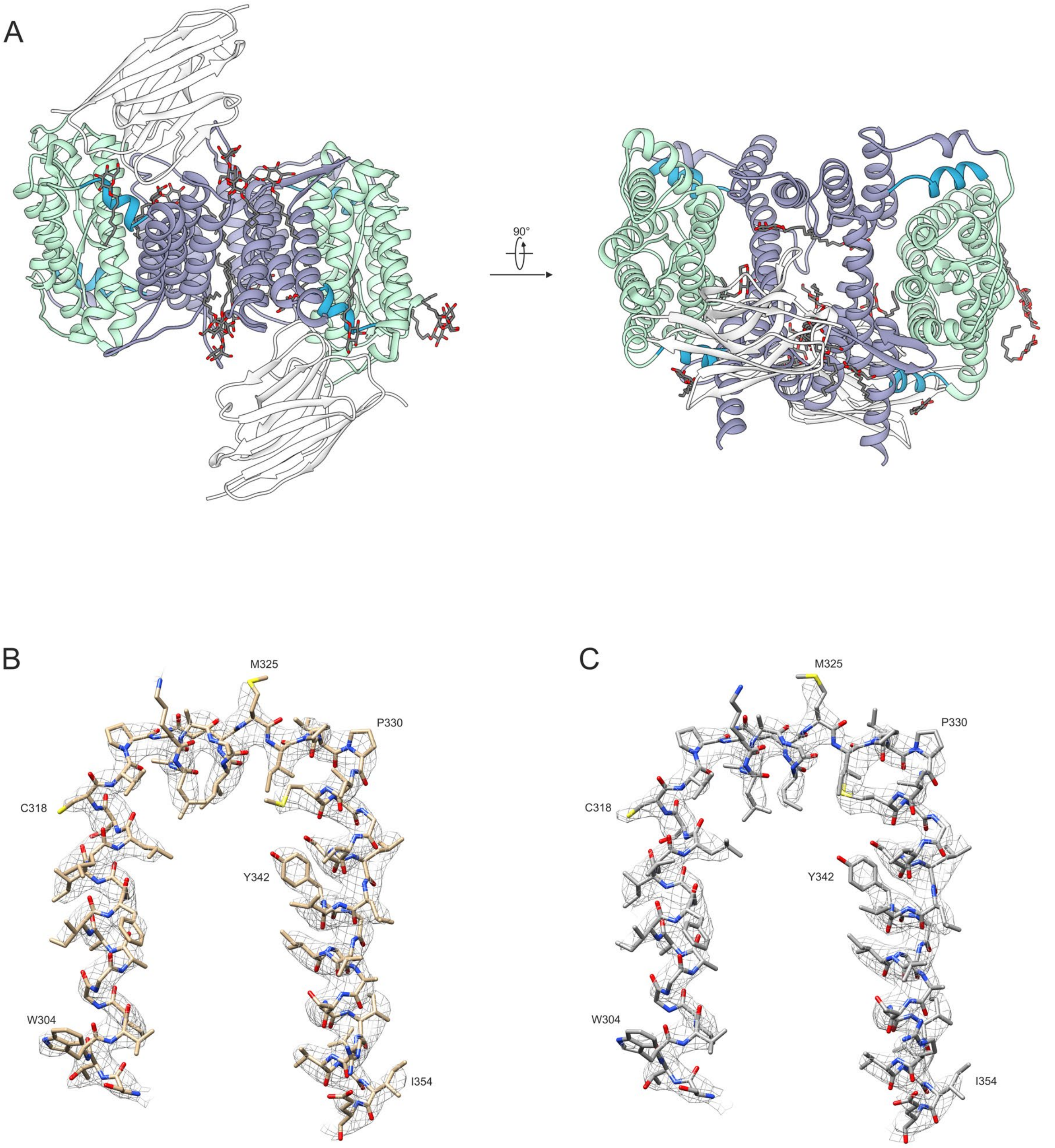

Supplementary Fig. 7: (A) Asymmetric unit of the UraA(G320P)-Sy45 crystal structure harboring two monomeric UraA protomers (purple/green) with Sy45 (grey) bound to a cytoplasmic epitope. Detergent molecules are visualized as sticks (dark grey). Side view from within the membrane (left panel) and top/bottom view (right panel). The UraA(G320P)-Sy45 crystal holds two monomeric UraA protomers in the asymmetric unit that show non-crystallographic symmetry allowing structural alignment with an RMSD of 0.36 Å. Though both protomers form crystal contacts via their scaffold domains, the assembly deviates from the native UraA dimer [13] with one protomer rotated by 160°. This arrangement avoids the steric overlap that both Sy45 binders in a dimer would experience. **(B)** Portion of the 2Fo-Fc electron density map of UraA(G320P)-Sy45 contoured at 1.5 σ. **(C)** Portion of the 2Fo-Fc electron density map of UraA(G320P)-Sy45 Uracil contoured at 1.5 σ.

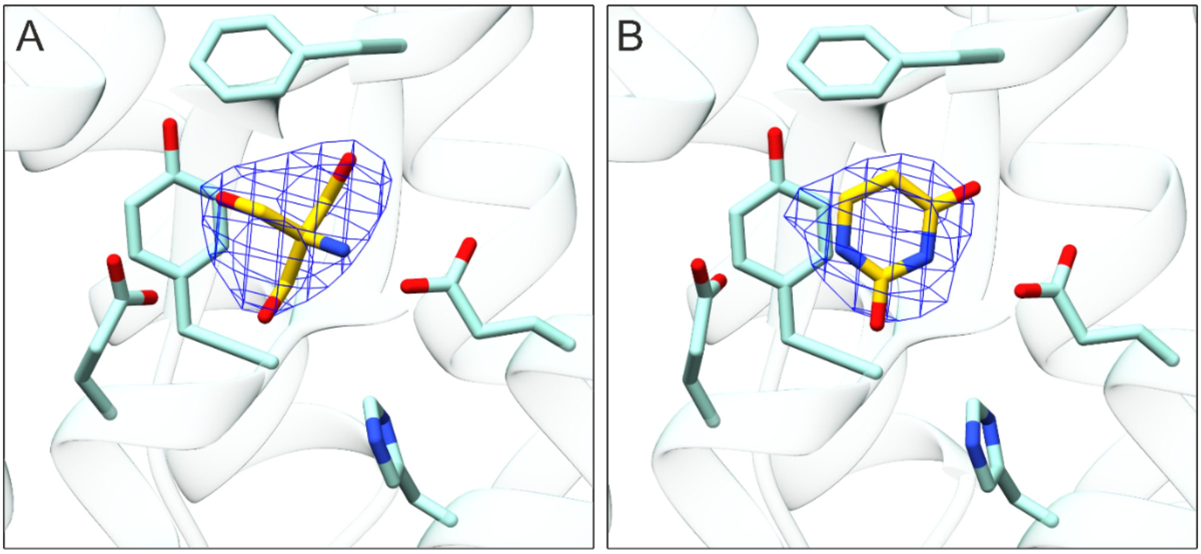

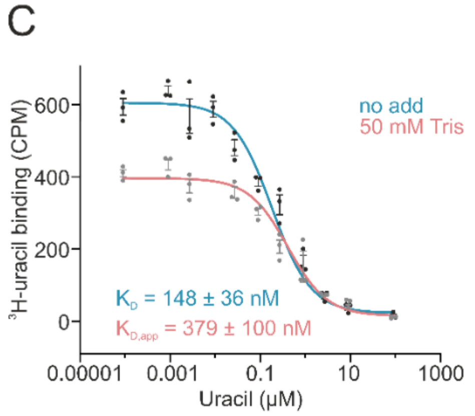

D

dataset	UraA(G320P)-Sy45	UraA(G320P)-Sy45 Uracil
$Uracil_0$ (mM)	-	1
K_D (nM)	144 ± 18	144 ± 18
$Tris_0$ (mM)	50	50
K_I (mM)	32 ± 20	32 ± 20
Uracil bound (%)	-	99.9 ± 1.8
Tris bound (%)	60.9 ± 14.8	0.022 ± 0.003

E

(1) $$K_{D,app} = K_D(1 + Tris/K_I)$$

(2) $$\frac{UraA_{Uracil}}{UraA_0} = \frac{(Uracil_0 + K_D(1 + Tris_0/K_I) + UraA_0) - \sqrt{(Uracil_0 + K_D(1 + Tris_0/K_I) + UraA_0)^2 - 4Uracil_0UraA_0}}{2UraA_0}$$

(3) $$\frac{UraA_{Tris}}{UraA_0} = \frac{(Tris_0 + K_I + UraA_0) - \sqrt{(Tris_0 + K_I + UraA_0)^2 - 4Tris_0UraA_0}}{2UraA_0}$$

Supplementary Fig. 8: Substrate binding site of UraA(G320P)-Sy45 apo **(A)** and uracil **(B)** structures. The Fo-Fc omit maps are shown as blue mesh and contoured at 3σ. Though the UraA(G320P)-Sy45 complex was co-crystallized in absence of uracil, additional electron density in the substrate binding site was observed. As substantial co-purification of uracil could be excluded based on the observed thermostabilization upon addition of uracil **[Fig. 1E]**, we modeled the buffer compound Tris into the density. **(C)** Scintillation proximity assay of UraA wild type in absence and presence of 50 mM Tris as indicated with three technical replicates shown as grey scatter and derived SER shown as black error bars. Scintillation data was fitted in Origin with a binding curve for homologous competition to calculate the dissociation constants. **(D)** Uracil- and Tris-bound fractions of UraA(G320P) present under the crystallization conditions as calculated with equations shown in **(E).** Based on the identical localization of the ligand electron density in the two structures we assumed Tris to be a competitive inhibitor. The K_I of Tris was calculated using equation (E1). Based on the affinity for uracil (K_D = 144 nM) and Tris (K_I = 32 mM) and the concentrations in the crystallization condition (uracil: 1 mM, Tris: 50 mM, UraA: 0.16 mM) the uracil-bound fraction was calculated with equation (E2) and exceeded 99%. The Tris bound fraction in absence of uracil was calculated to be 61 ± 15% with equation (E3).

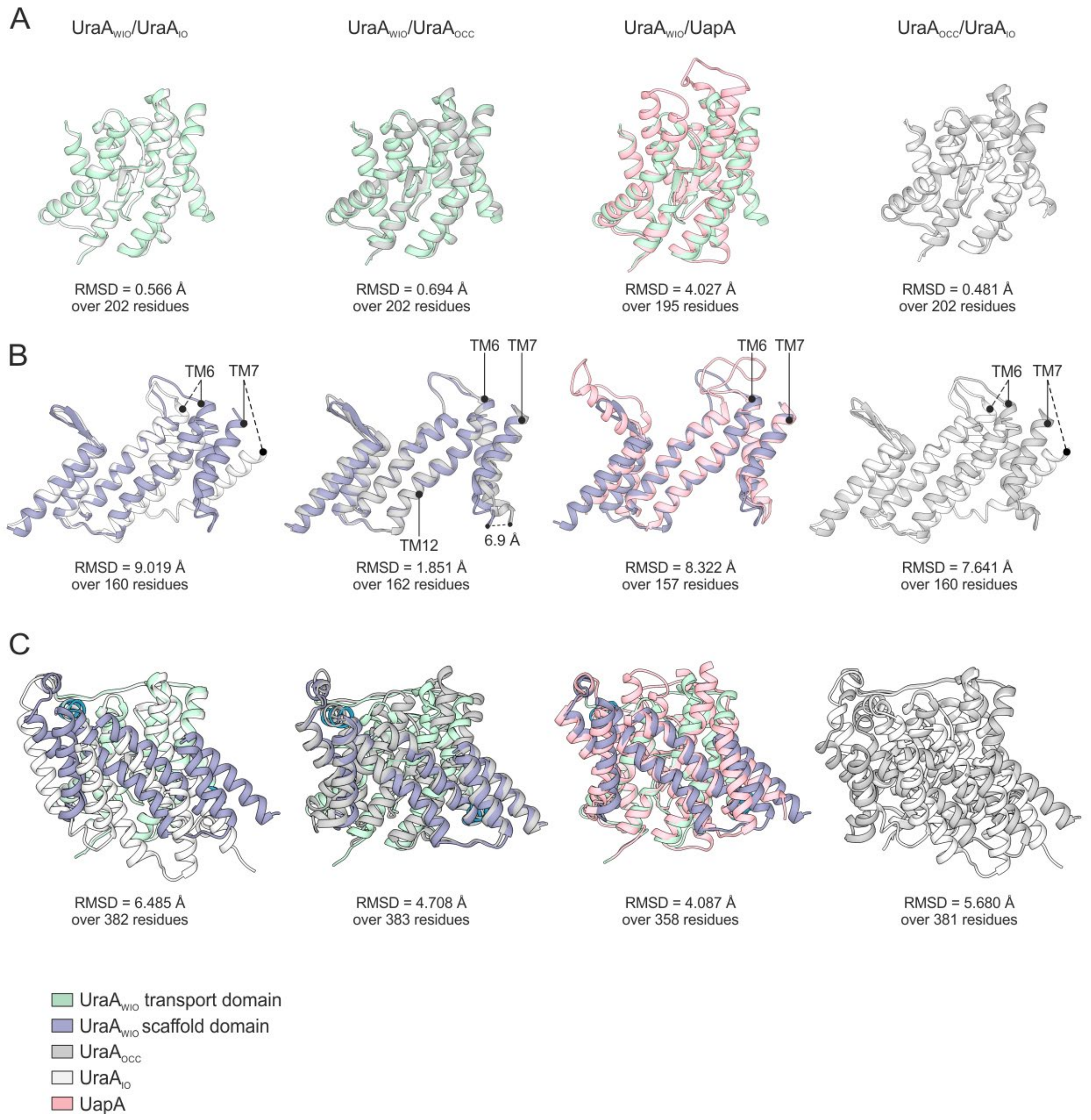

Supplementary Fig. 9: Structural alignments of SLC23 structures. Structural alignment of the transport **(A)** and scaffold domains **(B)** of relevant SLC23 structures using the default matchmaker command of UCSF Chimera [66]. **(C)** Whole protein structural alignment. For the alignment with UapA we truncated the TM2-3, TM3-4, TM5-6 and TM13-14 loops in UapA and the TM5-6 and TM13-14 loops in UraA to emphasize the high similarity in the orientation of the α-helices. Without these truncations an RMSD of 6.5 Å over 397 residues is obtained.

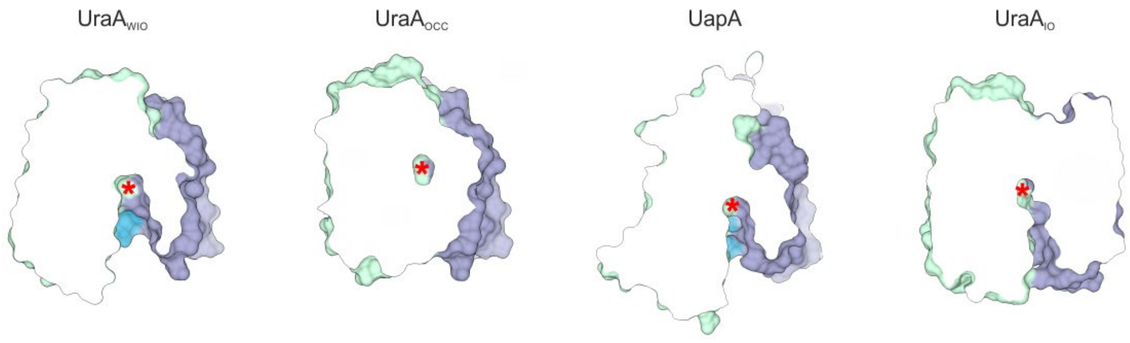

Supplementary Fig. 10: Cavity of $UraA_{WIO}$, $UraA_{OCC}$, $UraA_{IO}$ and UapA shown as side view from within the membrane cut through in the plane of the substrate binding site (red asterisk) with transport and scaffold domain in green and purple color, respectively.

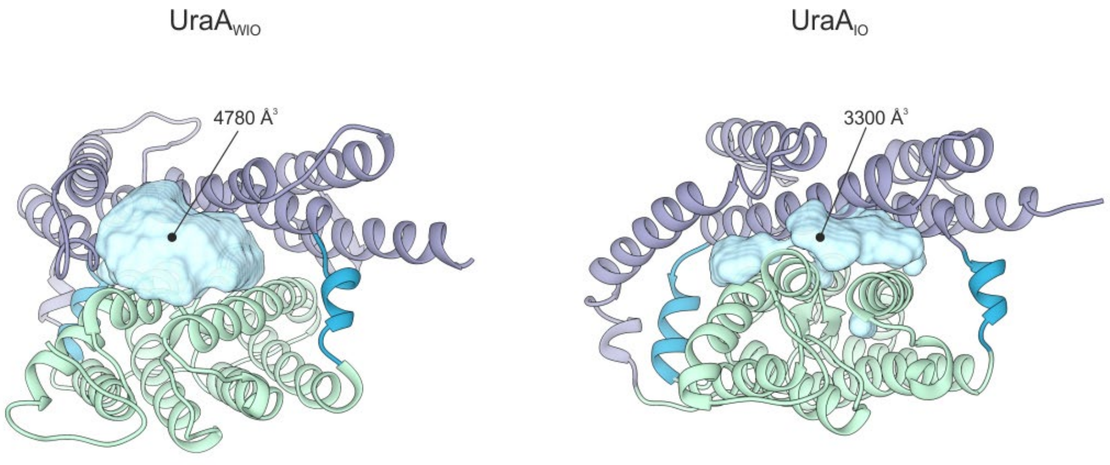

Supplementary Fig. 11: Cavity of UraA$_{WIO}$ and UraA$_{IO}$ shown as view from the cytoplasm with transport and scaffold domain in green and purple color, respectively. The water filled inward cavity generated with Hollow [36] is shown as blue sphere. Indicated volumes were calculated by the 3V webserver [37].

A

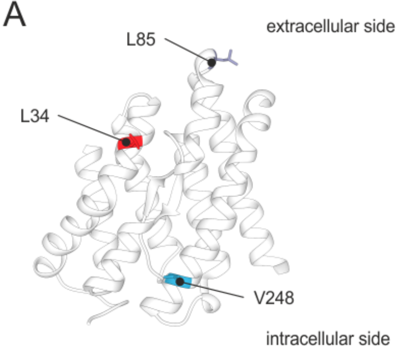

B

residue	$UraA_{WIO}$	$UraA_{WIO}$-Sy45	$UraA_{OCC}$	$UraA_{OO}$ (model)
L85	87.36 $Å^2$	87.36 $Å^2$	100.19 $Å^2$	110.84 $Å^2$
L34	0.64 $Å^2$	0.64 $Å^2$	7.43 $Å^2$	54.70 $Å^2$
V248	74.53 $Å^2$	71.28 $Å^2$	0.00 $Å^2$	0.00 $Å^2$

Supplementary Fig. 12: Solvent accessibility of conformational specific reporter positions. (A) Location of reporter positions in the transport domain of UraA. The scaffold domain was removed for better visualization. **(B):** Solvent accessible surface area of reporter positions in the relevant UraA structures and the outward-open model ($UraA_{OO}$).

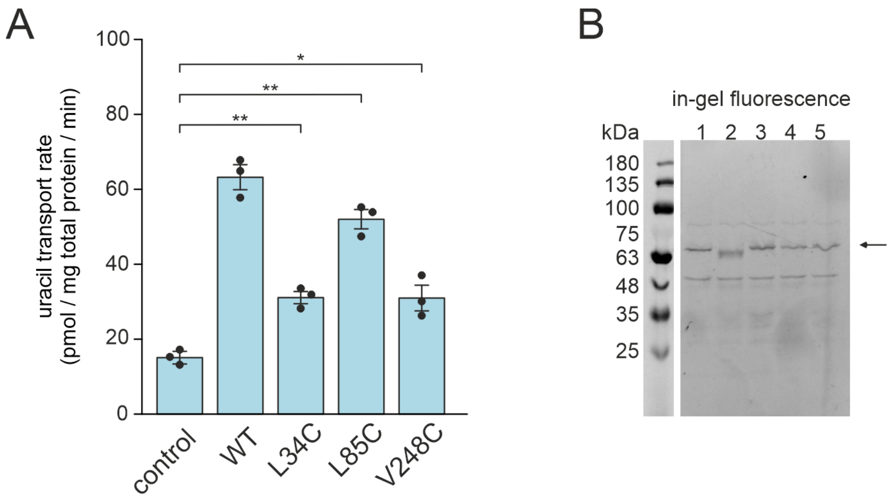

Supplementary Fig. 13: (A) Transport rates of [^{3}H]-uracil by UraA variants in *E. coli* BW25113(ΔuraA) with technical replicates (n=3 for all samples) shown as scatter plot and derived mean values ± SER as bars. **(B)** In-gel fluorescence of SDS-PAGE gel loaded with the samples derived from the cells used for the transport assay. Single cysteine mutants L34C, L85C, and V248C were generated in a cysteine-less background (UraA(C61S/C97S/C102S/C318A), hereafter referred to as UraA^{CL}). All UraA derivatives (1: UraA(WT); 2: control UraA(E241A/H245A/E290A); 3: UraA^{CL}(L34C); 4: UraA^{CL}(L85C); 5: UraA^{CL}(V248C)) were expressed from the pBXC3GH plasmid as a C-terminal GFP fusion protein.

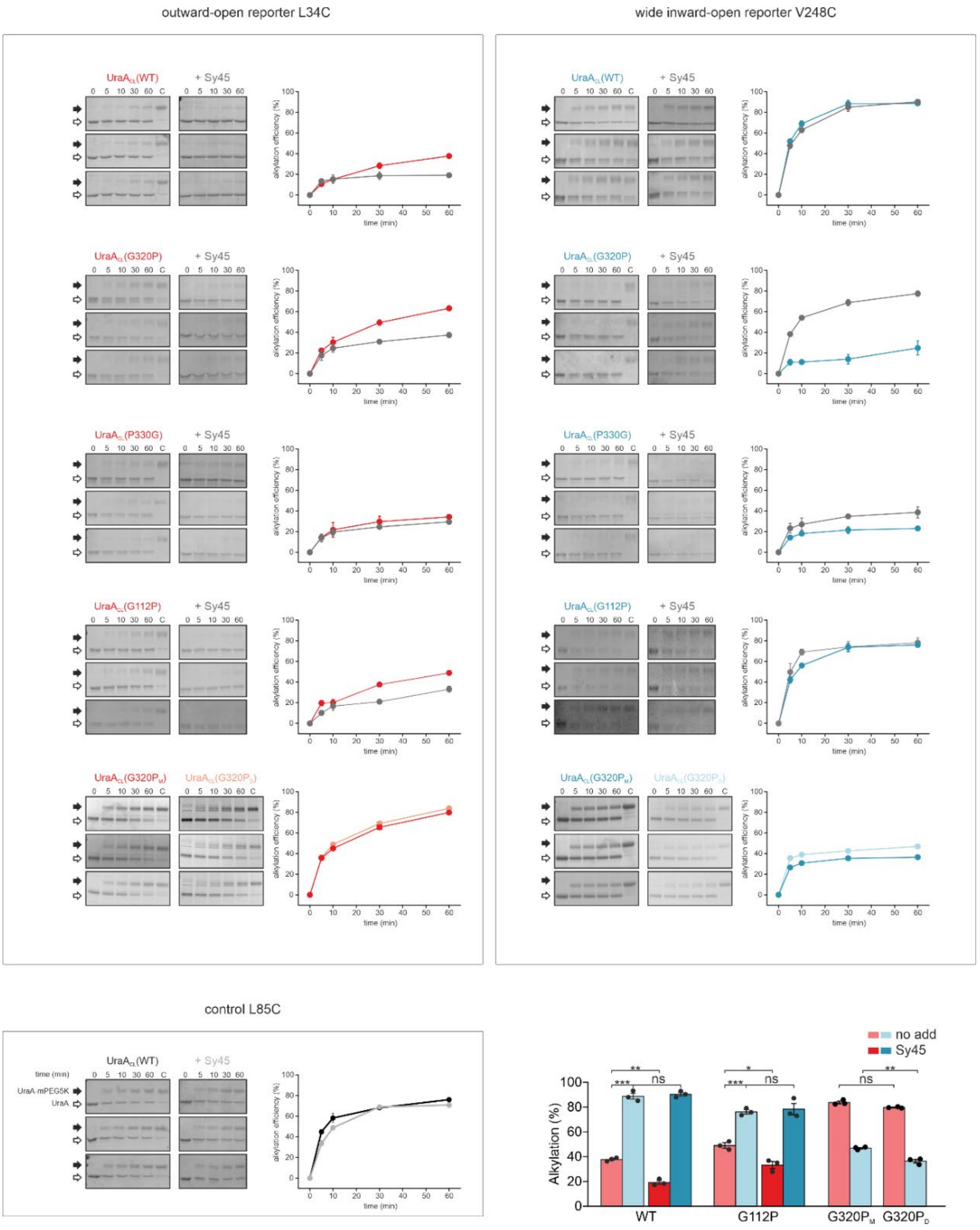

Supplementary Fig. 14: Degree of cysteine alkylation of L85C (black, control), L34C (red, outward-open reporter) and V248C (blue, wide inward-open reporter) in the presence and absence of Sy45 (grey). UraA variants were alkylated with mPEG5k over a time course of 1 hour and alkylation was quenched at indicated time points and quantified by mobility shift in SDS-PAGE and densitometry analysis. Data was normalized to the maximal alkylation obtained in the presence of 1% SDS (lane C). Shown are the mean of three technical replicates and standard error. For UraA(G320P) the monomer and dimer fractions from size-exclusion chromatography were analyzed seperately and depicted as $G320P_M$ and $G320P_D$. A two-tailed, unpaired t-test was performed to test for statistical significance (*$p < 0.05$, **$p < 0.01$, ***$p < 0.001$).

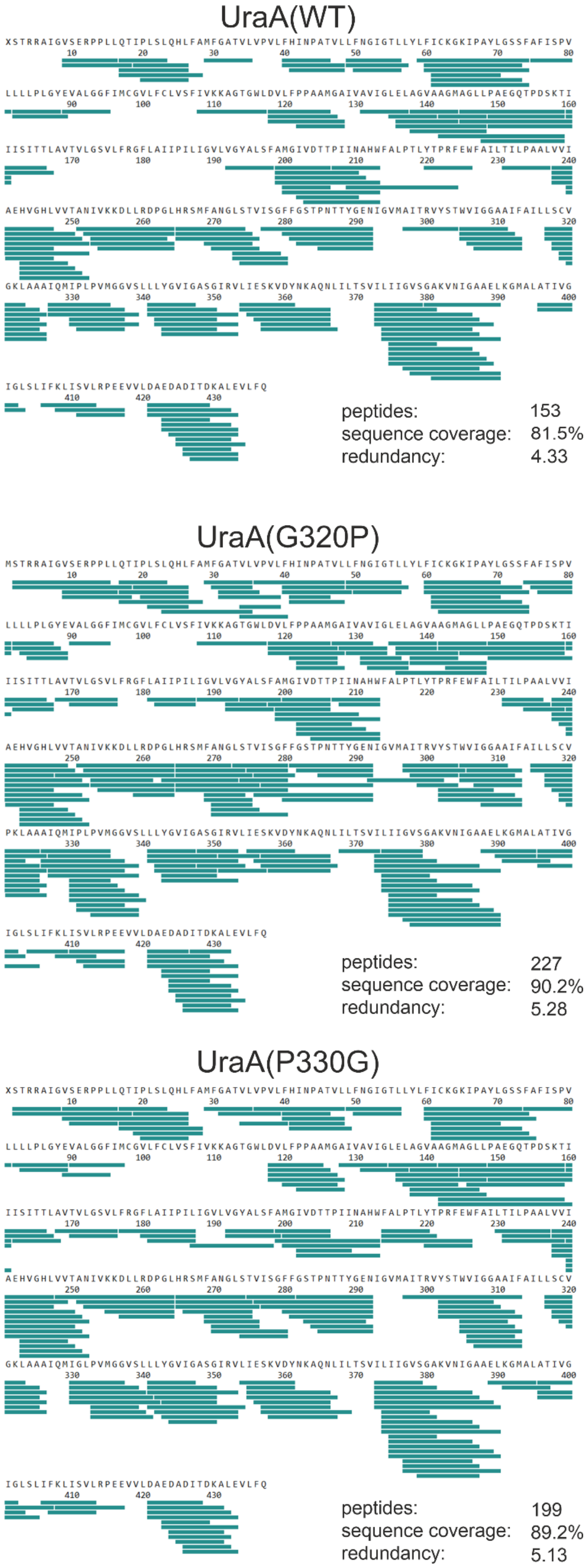

Supplementary Fig. 15: Sequence coverage maps of all UraA variants with its corresponding peptide numbers and redundancy. Data shown is the sequence coverage after data evaluation with DynamX. Peptides are shown in green bars. No statistical tests were applied to generate the coverage maps.

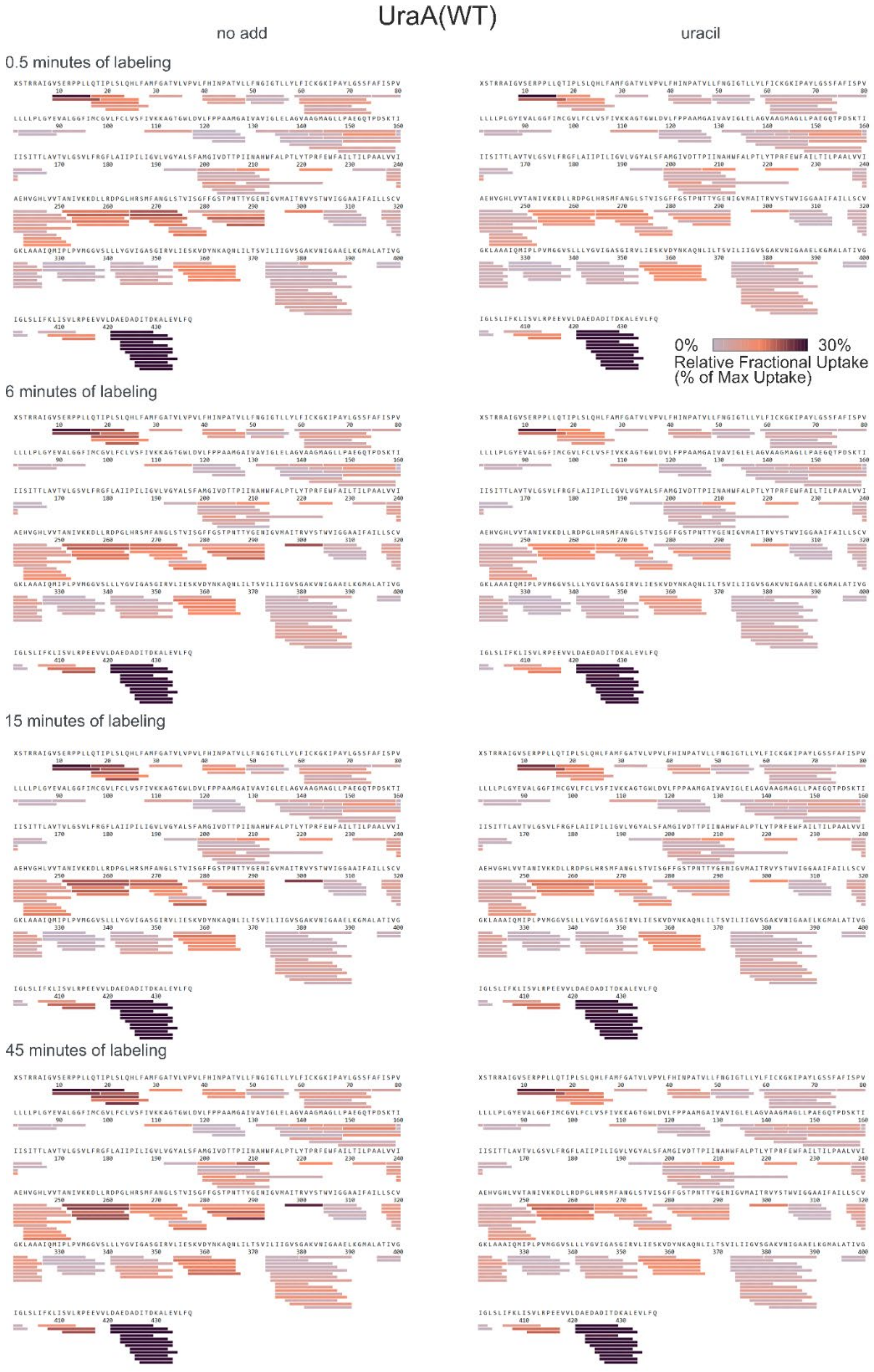

UraA(WT)
no add
uracil
0.5 minutes of labeling
0%
30%
Relative Fractional Uptake
(% of Max Uptake)
6 minutes of labeling
15 minutes of labeling
45 minutes of labeling

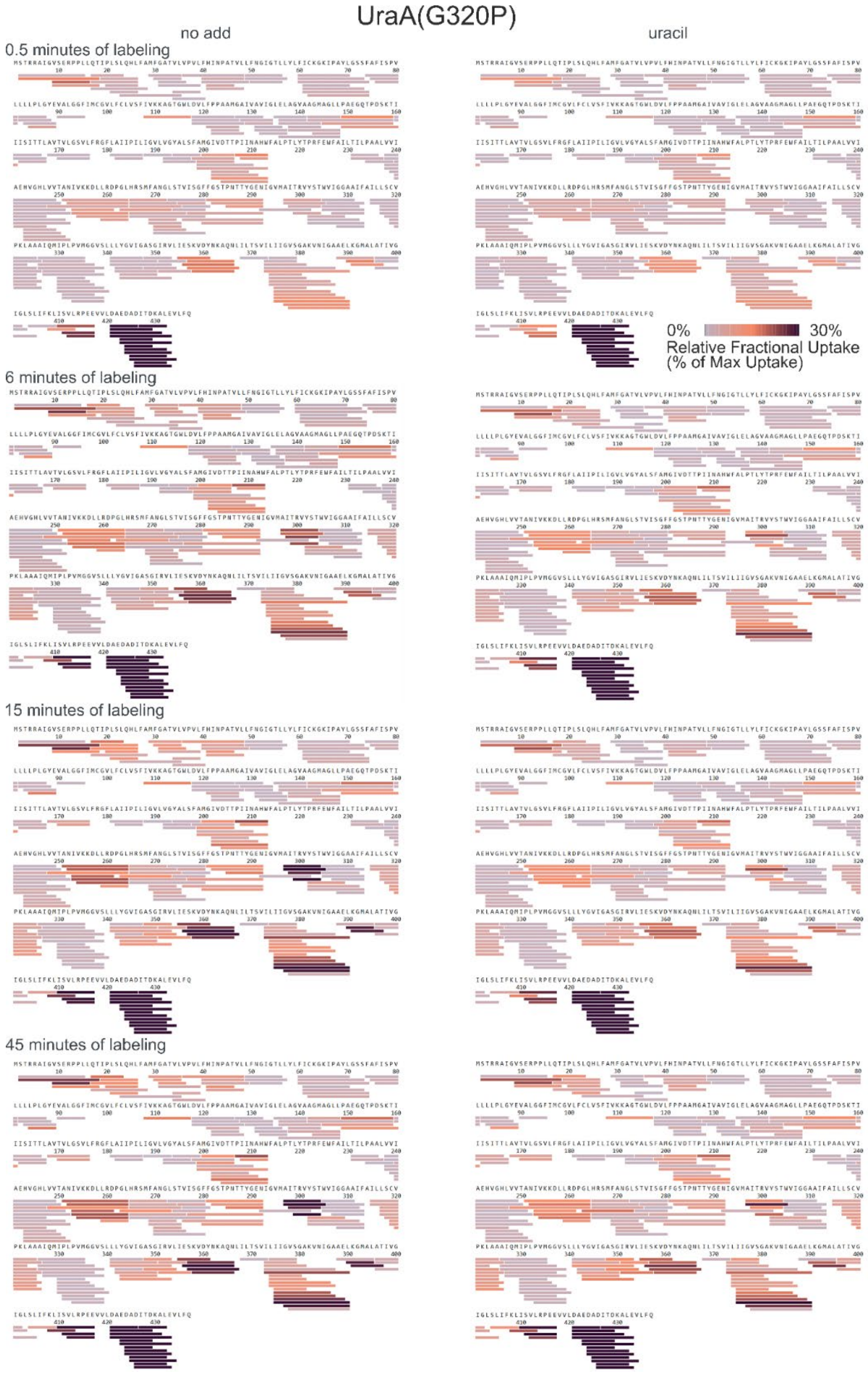

UraA(G320P)
no add
uracil
0.5 minutes of labeling
6 minutes of labeling
15 minutes of labeling
45 minutes of labeling
0%
30%
Relative Fractional Uptake
(% of Max Uptake)

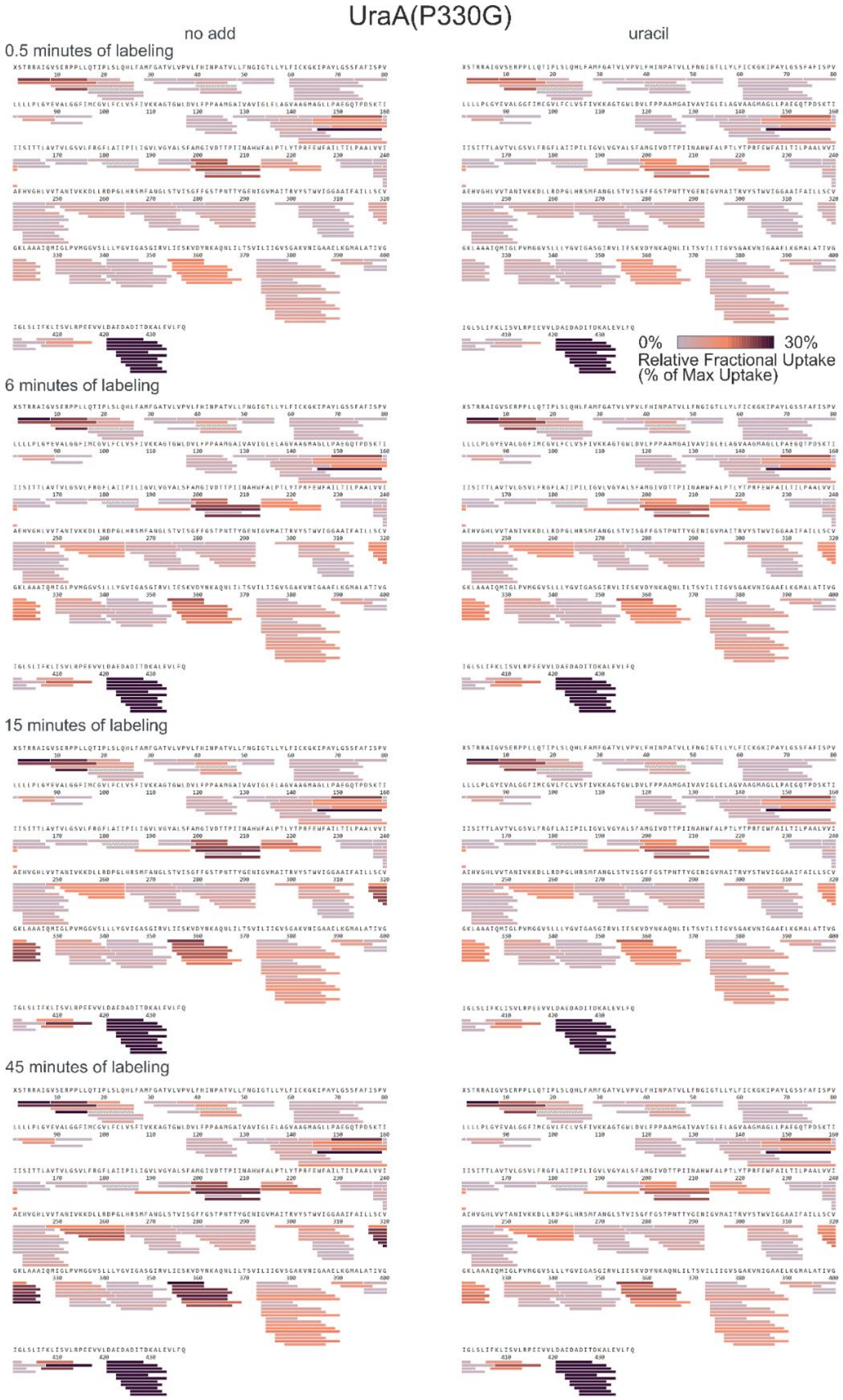

Supplementary Fig. 16: Relative deuterium uptake of UraA variants in presence and absence of uracil at 4 different time points (0.5 min, 6 min, 15 min and 45 min).

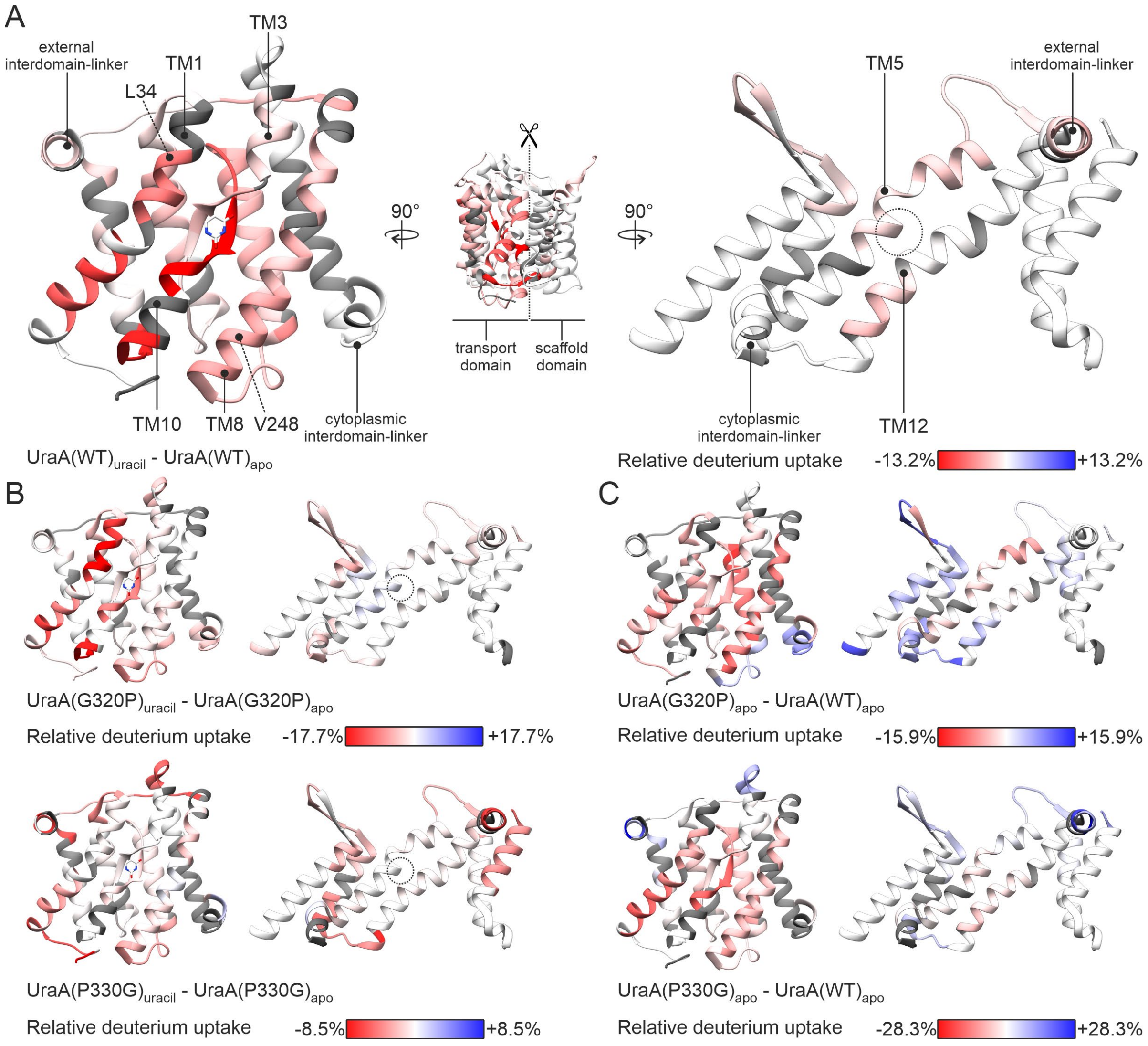

Supplementary Fig. 17: Hydrogen-deuterium exchange mass spectrometry analysis of UraA variants with scale bar adjusted to individual datasets. (A) HDX of wild type UraA in the presence and absence of 100 µM uracil. Transport and scaffold domain are oriented as if the protein was folded open as indicated in the central panel. The interdomain-linkers are shown in both domains as a reference point. The circle on the scaffold domain represents the position of the substrate in the opposing transport domain assuming the $UraA_{OCC}$ conformation. **(B)** Substrate-dependent HDX for UraA(G320P) and UraA(P330G). **(C)** Differential HDX resulting from the G320P (top panel) and P330G (lower panel) mutations in the absence of ligand compared to wild type UraA. The color range representing the relative deuterium uptake, ranging from red (decreased uptake) over white (no change) to blue (increased deuterium uptake), applies to all panels. Grey stretches indicate regions of not identified amino acids.

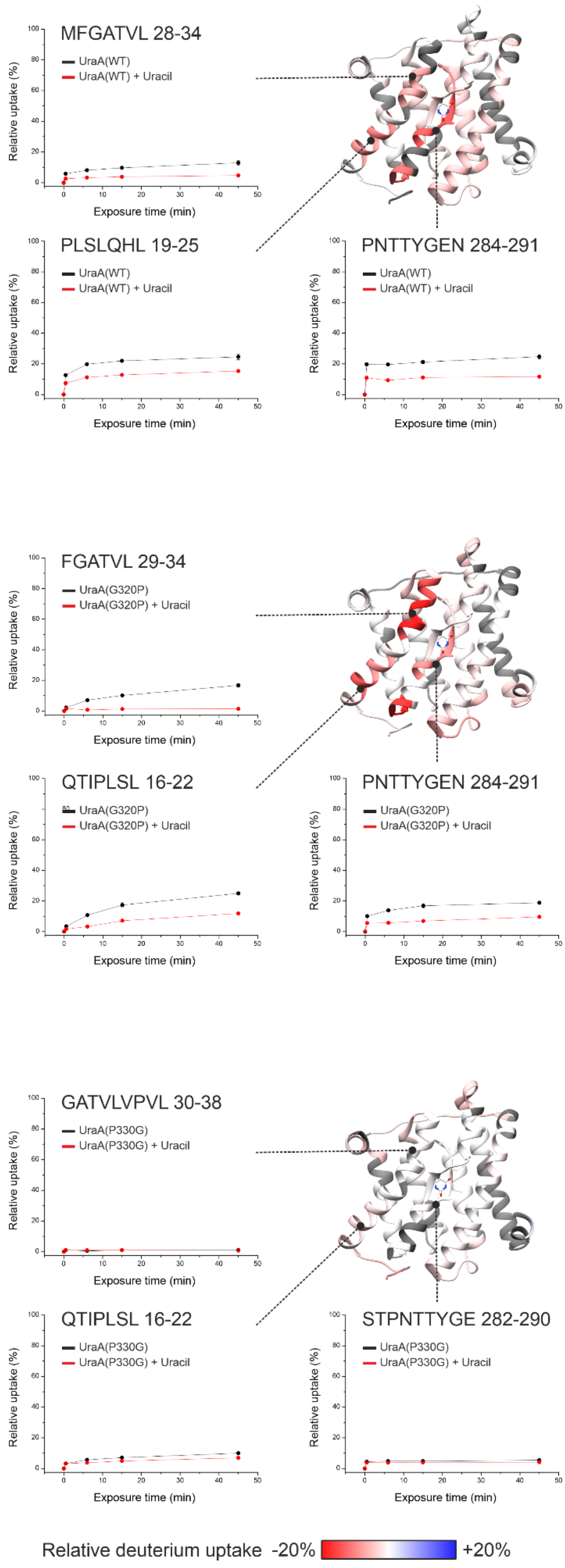

Supplementary Fig. 18: Relative deuterium uptake of 3 selected peptides for each UraA variant in presence and absence of uracil at four different time points (0.5 min, 6 min, 15 min and 45 min). Datapoints represent mean values ± SER from four technical replicates.
