# Supplementary material for: Interdomain-linkers control conformational transitions in the SLC23 elevator transporter UraA
Source: Nat Commun. 2024 Aug 30;15:7518. doi: 10.1038/s41467-024-51814-8 (PMC11362169; doi:10.1038/s41467-024-51814-8)
Supplement: Supplementary file 3 — Description of Additional Supplementary Files [file 41467_2024_51814_MOESM3_ESM.pdf]

Description of Additional Supplementary Files

File Name: Supplementary Data 1

Description: Primer sequences

File Name: Supplementary Data 2

Description: HDX data table for wild type UraA analyzed in the presence and absence of uracil.

File Name: Supplementary Data 3

Description: HDX data table for UraA(G320P) analyzed in the presence and absence of uracil

File Name: Supplementary Data 4

Description: HDX data table for UraA(P330G) analyzed in the presence and absence of uracil

File Name: Supplementary Data 5

Description: HDX data table for the comparison of apo-UraA(WT) and apo-UraA(G320P)

File Name: Supplementary Data 6

Description: HDX data table for the comparison of apo-UraA(WT) and apo-UraA(P330G)
