# Supplementary material for: Interdomain-linkers control conformational transitions in the SLC23 elevator transporter UraA
Source: Nat Commun. 2024 Aug 30;15:7518. doi: 10.1038/s41467-024-51814-8 (PMC11362169; doi:10.1038/s41467-024-51814-8)
Supplement: Supplementary file 10 — Reporting Summary [file 41467_2024_51814_MOESM10_ESM.pdf]

nature portfolio

Corresponding author(s): Eric R. Geertsma

Last updated by author(s): Jul 31, 2024

Reporting Summary

Nature Portfolio wishes to improve the reproducibility of the work that we publish. This form provides structure for consistency and transparency in reporting. For further information on Nature Portfolio policies, see our Editorial Policies and the Editorial Policy Checklist.

Statistics

For all statistical analyses, confirm that the following items are present in the figure legend, table legend, main text, or Methods section.

n/a	Confirmed	
☐	☒	The exact sample size (n) for each experimental group/condition, given as a discrete number and unit of measurement
☐	☒	A statement on whether measurements were taken from distinct samples or whether the same sample was measured repeatedly
☐	☒	The statistical test(s) used AND whether they are one- or two-sided<br>*Only common tests should be described solely by name; describe more complex techniques in the Methods section.*
☒	☐	A description of all covariates tested
☒	☐	A description of any assumptions or corrections, such as tests of normality and adjustment for multiple comparisons
☐	☒	A full description of the statistical parameters including central tendency (e.g. means) or other basic estimates (e.g. regression coefficient) AND variation (e.g. standard deviation) or associated estimates of uncertainty (e.g. confidence intervals)
☐	☒	For null hypothesis testing, the test statistic (e.g. F, t, r) with confidence intervals, effect sizes, degrees of freedom and P value noted<br>*Give P values as exact values whenever suitable.*
☒	☐	For Bayesian analysis, information on the choice of priors and Markov chain Monte Carlo settings
☒	☐	For hierarchical and complex designs, identification of the appropriate level for tests and full reporting of outcomes
☒	☐	Estimates of effect sizes (e.g. Cohen's d, Pearson's r), indicating how they were calculated

Our web collection on statistics for biologists contains articles on many of the points above.

Software and code

Policy information about availability of computer code

Data collection	MikroWin 300SL, 1450-3010 MicroBeta Workstation software, Rotor-Gene Q 2.1.0.9, ImageQuant LAS 4000, Mass Lynx 4.2, PrepCon 5.09.115
Data analysis	PHENIX 1.17.1-3660-000, XDS, PDB_EXTRACT 3.27, Chimera 1.14, WinCoot 0.9.8.1, ImageJ 1.52a, OriginPro 8.6, CorelDraw X7, DynamX 3.0, R-Studio, Microsoft Excel, Hollow 1.3

For manuscripts utilizing custom algorithms or software that are central to the research but not yet described in published literature, software must be made available to editors and reviewers. We strongly encourage code deposition in a community repository (e.g. GitHub). See the Nature Portfolio guidelines for submitting code & software for further information.

Data

Policy information about availability of data

All manuscripts must include a data availability statement. This statement should provide the following information, where applicable:

- Accession codes, unique identifiers, or web links for publicly available datasets
- A description of any restrictions on data availability
- For clinical datasets or third party data, please ensure that the statement adheres to our policy

The source data underlying Figures 1D, 1F, 4B-D, and Supplementary Figures 13A and 14 are provided as Source Data File. The atomic coordinates have been deposited in the Protein Data Bank under accession code 8OMZ (https://doi.org/10.2210/pdb8omz/pdb; unliganded UraA(G320P)-Sy45) and 8OO1 (https://doi.org/10.2210/pdb8oo1/pdb; liganded UraA(G320P)-Sy45). The previously published UraA structure in an occluded conformation is accessible under the

accession code 5XLS (http://doi.org/10.2210/pdb5xls/pdb). HDX-MS data tables are provided as Supplementary Data 2-6. The complete HDX-MS dataset including raw spectra has been deposited to the ProteomeXchange Consortium via the PRIDE partner repository with the dataset identifier PXD054394.

Research involving human participants, their data, or biological material

Policy information about studies with human participants or human data. See also policy information about sex, gender (identity/presentation), and sexual orientation and race, ethnicity and racism.

Reporting on sex and gender	not applicable
Reporting on race, ethnicity, or other socially relevant groupings	not applicable
Population characteristics	not applicable
Recruitment	not applicable
Ethics oversight	not applicable

Note that full information on the approval of the study protocol must also be provided in the manuscript.

Field-specific reporting

Please select the one below that is the best fit for your research. If you are not sure, read the appropriate sections before making your selection.

☒ Life sciences ☐ Behavioural & social sciences ☐ Ecological, evolutionary & environmental sciences

For a reference copy of the document with all sections, see nature.com/documents/nr-reporting-summary-flat.pdf

Life sciences study design

All studies must disclose on these points even when the disclosure is negative.

Sample size	Sample size estimation was not relevant for this study, as it does not report on a statistical evaluation of effects between two or more groups.
Data exclusions	No data were excluded from the analysis
Replication	Uracil transport (Fig. 1D) was carried out with technical replicates (n=3 for UraA(G112P), UraA(G320P), UraA(P121G), UraA(P330G); n=4 for UraA(WT); n=6 for control). This data was used to calculate mean and standard errors (Fig. 1D).<br><br>Thermal shift assay was carried out with technical replicates (n=3 for samples without Sy45, n=4 for samples with Sy45). Mean and standard deviation of individual melting curves are presented (Fig. S5, Fig. S6).<br>Mean and standard deviation of calculated melting temperatures are shown as bar graph with error bar (Fig. 1F, Fig. 4C).<br>A two-tailed, unpaired t-test was performed to test for statistical significance for UraA(P330G) (*p<0.05).<br><br>Cysteine alkylation assay (Fig. 4B) was carried out with technical replicates (n=3) for all samples.<br>Data was normalized to the maximal alkylation obtained in the presence of 1% SDS.<br>Individual data points are shown as scatter, mean and standard error as bar graph with error bar.<br>A two-tailed, unpaired t-test was performed to test for statistical significance (*p<0.05, **p<0.01, ***p<0.001).<br>All SDS-gels used are shown in Fig. S14.<br><br>Uracil transport (Fig. S13A) was carried out with technical replicates (n=3) for all samples. This data was used to calculate mean and standard errors (Fig. S13A).<br><br>Scintillation proximity assay was carried out with technical replicates (n=3) for all samples. Individual data points are shown as scatter (Fig. 4D). Calculated Mean was removed from the figure for better visualization. Standard errors are shown as error bars.
Randomization	No randomization was performed.
Blinding	Experiments were not blinded.

Reporting for specific materials, systems and methods

We require information from authors about some types of materials, experimental systems and methods used in many studies. Here, indicate whether each material, system or method listed is relevant to your study. If you are not sure if a list item applies to your research, read the appropriate section before selecting a response.

Materials & experimental systems

n/a	Involved in the study
☐	☒ Antibodies
☒	☐ Eukaryotic cell lines
☒	☐ Palaeontology and archaeology
☒	☐ Animals and other organisms
☒	☐ Clinical data
☒	☐ Dual use research of concern
☒	☐ Plants

Methods

n/a	Involved in the study
☒	☐ ChIP-seq
☒	☐ Flow cytometry
☒	☐ MRI-based neuroimaging

Antibodies

Antibodies used: Sy45, synthetic nanobody selected in this study

Validation: crystal structure of Sy45 in complex with UraA(G320P)

Plants

Seed stocks: *Report on the source of all seed stocks or other plant material used. If applicable, state the seed stock centre and catalogue number. If plant specimens were collected from the field, describe the collection location, date and sampling procedures.*

Novel plant genotypes: *Describe the methods by which all novel plant genotypes were produced. This includes those generated by transgenic approaches, gene editing, chemical/radiation-based mutagenesis and hybridization. For transgenic lines, describe the transformation method, the number of independent lines analyzed and the generation upon which experiments were performed. For gene-edited lines, describe the editor used, the endogenous sequence targeted for editing, the targeting guide RNA sequence (if applicable) and how the editor was applied.*

Authentication: *Describe any authentication procedures for each seed stock used or novel genotype generated. Describe any experiments used to assess the effect of a mutation and, where applicable, how potential secondary effects (e.g. second site T-DNA insertions, mosiacism, off-target gene editing) were examined.*
